# Socially desirable responding in geriatric outpatients with and without mild cognitive impairment and its association with the assessment of self-reported mental health

**DOI:** 10.1186/s12877-021-02435-z

**Published:** 2021-09-15

**Authors:** Paola Nicolini, Carlo Abbate, Silvia Inglese, Daniela Mari, Paolo D. Rossi, Matteo Cesari

**Affiliations:** 1 Geriatric Unit, Fondazione IRCCS Ca’ Granda Ospedale Maggiore Policlinico, Milan, Italy; 2 IRCCS Fondazione don Carlo Gnocchi, Milan, Italy; 3 Università degli Studi di Milano, Milan, Italy

**Keywords:** Social desirability, Older adults, Geriatric outpatients, Mild cognitive impairment, Self-reported mental health, Depressive symptoms, Anxiety symptoms, Marlowe-Crowne social desirability scale

## Abstract

**Background:**

Socially desirable responding is a potentially relevant issue in older adults and can be evaluated with the Marlowe-Crowne Social Desirability Scale (MCSDS). However, the eight-item MCSDS has never been specifically administered to geriatric subjects, and there is a dearth of literature on the relationship between social desirability and cognitive impairment. Also, the connection between social desirability and subjective measures of psychological well-being is a matter of controversy. This study has three main aims. First, to determine the psychometric properties of the eight-item MCSDS in geriatric outpatients without dementia (i.e. with normal cognition (NC) or mild cognitive impairment (MCI)). Second, to investigate the link between social desirability and cognitive functioning. Third, to determine the association between social desirability and the assessment of self-reported mental health.

**Methods:**

Community-dwelling outpatients (aged ≥ 65) were consecutively recruited and neuropsychologically tested to diagnose NC or MCI (*n* = 299). Social desirability was assessed with the eight-item MCSDS. Depressive and anxiety symptoms were measured with the short Geriatric Depression (GDS-s) and the State-Trait Personality Inventory Trait Anxiety (STPI-TA) scales.

**Results:**

On principal components analysis, the eight-item MCSDS was found to have a multidimensional structure. Of the initial three-component solution, only two subscales had acceptable internal consistency (Cronbach’s alpha > 0.6): “Acceptance of responsibility” and “Integrity”. The third subscale (“Kindness towards others”) appeared to gauge two distinct constructs of formal (i.e. politeness) versus substantive (i.e. forgiveness) compassion. On binary logistic regression, only higher income was a significant predictor of formal compassion. Test-retest reliability was substantial to excellent (Gwet’s AC2 ≥ 0.8). There were no meaningful differences in social desirability between the NC and MCI groups. Likewise, negative Spearman’s correlations between social desirability and cognitive Z-scores across the whole sample were weak (r_s_ < |0.3|) and confined to one MCSDS item. Although social desirability was an independent predictor of the STPI-TA score in multiple linear regression, it explained only a marginal amount of incremental variance in anxiety symptoms (less than 2%).

**Conclusions:**

Our results suggest that social desirability need not be a major concern when using questionnaires to assess mental health in geriatric outpatients without dementia.

**Supplementary Information:**

The online version contains supplementary material available at 10.1186/s12877-021-02435-z.

## Background

Social desirability is the tendency of subjects to respond to self-report items in a manner that portrays them in an overly favourable light with respect to prevailing social norms and standards [1]. It can therefore affect all self-rated measures, especially those involving socially sensitive topics like psychological well-being [2, 3].

There are several lines of evidence that support the notion that socially desirable responding may be an important issue among older individuals. First, geriatric clinical practice and ageing research rely heavily on self-reports [4]. Second, it is acknowledged that social desirability increases with age (e.g. [5–9]), be it because older people compensate for the negative way ageing is stereotyped in our society by presenting a more positive image of themselves [10] or because they have more traditional values and are more sensitive to socially accepted norms [7]. Third, mild cognitive impairment (MCI) is highly prevalent in geriatric populations [11] and it could influence social desirability in two opposite directions. On the one hand, the stigma associated with the condition [12] could make subjects more prone to strategic self-presentation [10], thereby increasing social desirability. On the other hand, deficits in social cognitive abilities [13, 14] could make subjects less aware of common social norms, thereby reducing social desirability.

The most widely used tool to assess socially desirable responding is the Marlowe-Crowne Social Desirability Scale (MCSDS) [15] whose shorter versions (e.g. [16]) minimise time and fatigue and can therefore be better suited to the setting of a comprehensive geriatric assessment.

There are a number of gaps in the literature. The eight-item MCSDS used by Ray and coworkers [16] has never been specifically administered to subjects in the geriatric age range (i.e. aged 65 or more). Also, we are aware of only one study that has investigated the relationship between social desirability and measures of cognition. Indeed, Dijkstra et al. [7] report a negative correlation between the two, but their results may be very specific to the scale employed. In fact, while most short forms of the MCSDS are balanced in terms of positively- and negatively-keyed items [17], they have used the 12-item Eysenck Lie Scale which is composed of mainly (75%) negatively-keyed items (i.e. items for which disagreement indicates socially desirable responding). This leads the authors to hypothesise that poorer cognitive performance produces greater socially desirable responding because difficulties in retrieving information from memory make the respondents more likely to give a “no” answer. Lastly, the extent to which social desirability can impact self-reported psychological data, and hence the assessment of emotional well-being, is still a matter of debate, with some studies reporting an effect [8, 18–20] and others reporting none [21–26].

### Aims

The current study has three aims:

- To evaluate the psychometric properties (internal consistency, unidimensionality and test-retest reliability) of a short, eight-item, form of the MCSDS [16] when applied to geriatric outpatients without dementia (i.e. with normal cognition (NC) or MCI).
- To investigate the relationship between social desirability and cognitive functioning.
- To determine the association between social desirability and self-reported symptoms of depression and anxiety.

## Methods

### Participants

In this cross-sectional study we enrolled 359 community-dwelling older subjects (aged ≥ 65), without a known diagnosis of dementia, who consecutively attended a first geriatric visit at the Geriatric Outpatient Unit of our university hospital in Milan, Italy, from January 2018 to January 2019. After a general assessment, participants were invited to undergo an on-site neuropsychological evaluation. 299 subjects diagnosed with NC (*n* = 117) or MCI (*n* = 182) were administered the three scales (MCSDS and the scales for depressive and anxiety symptoms, see later) one month after the diagnosis. The scales were administered during a one-to-one interview with a geriatrician. In accordance with previous research [20–23], statements from the questionnaires were read to the respondent in order to facilitate understanding and minimise fatigue. The order of the scales was counterbalanced across participants to control for order effects.

To determine test-test reliability a random sample of 50 subjects were administered the MCSDS a second time, one month after the first administration. The size of this subsample was based on the recommendation that at least 30 subjects are required for reliability studies [27]. The time interval was chosen because it is the one originally used by Crowne and Marlowe [15] and it appears to strike a reasonable balance between the need to minimise recollection bias while ensuring clinical stability.

### General assessment

Information was collected on the sociodemographic characteristics of the participants: age, sex, education and income. Since income is a sensitive variable, participants were only asked to disclose if their monthly income was above or below the 1500 euro threshold, which is the reported mean for Italian pensioners [28]. The Mini Mental State Examination (MMSE) [29], corrected for age and education, was used to provide a crude measure of global cognition. Functional status was evaluated with the scales for the Basic Activities of Daily Living (BADL) [30] and the Instrumental Activities of Daily Living (IADL) [31]. Comorbidity was quantified by the Cumulative Illness Rating Scale comorbidity (CIRS-m) score [32].

### Neuropsychological assessment

The neuropsychological assessment was carried out by means of a comprehensive battery of tests investigating different cognitive domains: attention [33, 34], memory [35], executive functions [34, 36–40], language [34, 41], visuospatial skills [34, 42] and ideomotor praxis [43]. The neuropsychological tests are reported in Table 1. We computed a global cognitive Z-score for use as a fine-grained measure of cognitive function. The raw score from each neuropsychological test was transformed to a Z-score, based on the mean and standard deviation of the normative score distribution, and scores that quantified response time or number of errors were multiplied by − 1 so that lower Z-scores always indicated poorer performance. The Z-scores were then averaged across all tests to generate a composite. Since the medial temporal lobe (involved in memory) and the prefrontal cortex (involved in attention and executive functioning) have been shown to be neural substrates of social cognition [14], we also generated separate Z-scores for memory and attention/executive functioning.

**Table 1:** Neuropsychological tests and cognitive domains assessed

Cognitive domain	Neuropsychological test
Attention	Bell Test
	Digit Cancellation Test
Memory	Prose recall
	ROCF-delayed recall
Executive functions	Digit Span Forwards
	Digit Span Backwards
	Trail-Making Test A
	Trail-Making Test B
	Weigl’s Test
	Cognitive Estimates-total
	Cognitive Estimates-bizarre
	Raven’s coloured matrices
	Letter fluency
Language	Category fluency
	Picture naming
	Token Test
Visuospatial skills	ROCF-copy
	Copy of geometric figures
Ideomotor praxis	De Renzi’s Test - right upper limb
	De Renzi’s Test - left upper limb

**Legend**

Abbreviations: *ROCF* Rey-Osterrieth Complex Figure. For all tests higher scores indicate better cognitive functioning, except for the Trail-Making and Cognitive Estimates tests for which the reverse applies

MCI was diagnosed according to current consensus criteria of objective cognitive impairment on neuropsychological testing, essentially preserved daily functioning (i.e. intact BADL with no or minimal impairment of IADL) and no dementia [44]. Objective cognitive impairment was defined by at least one neuropsychological test having a score below the 10th percentile of the normative score distribution [45, 46].

### Assessment of social desirability

Social desirability was assessed with a short version of the MCSDS [16]. It consists of eight statements in question form describing socially desirable but uncommon behaviours (e.g. being always polite) and socially undesirable but common behaviours (e.g. being sometimes unforgiving). There are four positively-keyed and four negatively-keyed statements. A “yes” answer is scored 1 for negatively-keyed statements and 3 for positively-keyed statements. A “no” answer is reverse scored. A “don’t know” or missing answer is scored 2. Hence, scores range from 8 to 24, with higher scores indicating greater social desirability. The eight-item MCSDS can be found in Additional file 1.

### Assessment of depressive and anxiety symptoms

Depressive symptoms were assessed with the short, 15-item, form of the Geriatric Depression Scale (GDS-s) [47]. Answers are in a yes/no format and scores range from 0 to 15, with higher scores indicating greater depressive symptoms.

Anxiety symptoms were assessed with the Trait Anxiety (TA) scale from Spielberger’s State-Trait Personality Inventory (STPI) (STPI-TA) [48]. Answers are given on a 4-point Likert scale for the frequency of symptoms and scores range from 10 to 40, with higher scores indicating greater anxiety symptoms.

Both scales have been extensively validated in geriatric populations (e.g. [49, 50]), also including subjects with MCI (e.g. [51–54]).

### Statistical analysis

The statistical analysis was performed by means of the statistical packages SPSS version 26.0 (SPSS Inc., Chicago, IL) and R version 3.5.2 (The R Foundation for Statistical Computing, Vienna, Austria) for Windows. Parametric and non-parametric statistics were chosen as appropriate. Normality was assessed by visual inspection of QQ plots; linearity and homoscedasticity were assessed by visual inspection of residual versus predictor plots; lack of multicollinearity was assessed by the Variance Inflation Factor (VIF < 5); independence of errors was assessed by the Durbin-Watson test (values 1.97–2.06). The NC and MCI groups were compared by means of Student’s t-test or Mann-Whitney’s U-test for continuous variables and by means of the Chi-squared test for categorical variables. The internal consistency of the MCSDS was quantified by Cronbach’s alpha and by the average inter-item correlation. The latter was specifically evaluated since it is considered to be a better marker of internal consistency [55, 56]. In fact, it is recognised that Cronbach’s alpha depends on both the average inter-item correlation and the number of items in the scale [57], so that it can be high for lengthy scales with weak inter-item correlations and relatively low for short scales with stronger inter-item correlations. The factorial structure of the MCSDS was explored by conducting a principal components analysis (PCA) with an oblique rotation (direct oblimin), which is the most conservative since it allows for correlations between factors. The Kaiser-Meyer-Olkin measure of sampling adequacy (> 0.5) and Bartlett’s test of sphericity (< 0.05) indicated that the data were suitable for PCA. Components were extracted according to Kaiser’s criterion (eigenvalue > 1) [58]. Factor loadings were considered significant if they had an absolute value ≥ 0.60 [59, 60]. Test-retest reliability was evaluated with Gwet’s agreement coefficient (AC) which is a chance-corrected agreement statistic that, unlike Cohen’s kappa, does not underestimate reliability when there is a high prevalence of one response category [61, 62]. In particular, linear-weighted Gwet’s AC2 for interval data was used [61–64]. Although the correlation coefficient is a suboptimal measure of test-retest reliability because it does not assess the extent of agreement between variables and as such is vulnerable to systematic bias [65, 66], the test-retest reliability of the total MCSDS score was also calculated with Spearman’s correlation for comparison with other studies [67]. The relationship between the MCSDS and cognitive function scores was evaluated with Spearman’s simple and partial correlations. The association between social desirability and psychological symptoms was investigated by means of Spearman’s simple correlations and multiple linear regression. Sociodemographic and clinical characteristics were considered potential confounders and adjusted for in correlation and regression analyses. A *p* value ≤ 0.05 was taken to be statistically significant. Because of the exploratory nature of the study we did not correct for multiple testing [68].

A power determination analysis was conducted for the current sample size with G*power software [69] for Mann-Whitney’s U-test and multiple linear regression, and with the formula by Bonett and Wright for Spearman’s correlations [70]. In order to be conservative, given the novelty of the study, we assumed small expected effects sizes (r = 0.2, f^2^ = 0.04). The achieved sample size of almost 300 participants had a 93% power, with a 5% alpha level (two-tailed), for both Spearman’s correlations and multiple linear regression. Also, considering a minimal clinically significant difference of 1.7 points on the MCSDS [71], based on the standard deviation of previous data from Ray and Lovejoy [72] on the administration of the eight-item MCSDS to subjects in the fifth age decade, Mann-Whitney’s U-test had a 98% power, with a 5% alpha level (two-tailed), to detect a difference between the two groups.

With regard to the test-retest sample size, a power determination was performed with G*power, based on the reported test-retest reliability coefficients for different versions of the MCSDS (r = 0.4 to 0.9) [67]. Considering the lowest value as the most conservative, the *n* = 50 sample size provided 90% power with a 5% alpha level (one-tailed).

## Results

### Sample characteristics

Table 2 summarises the main sociodemographic and clinical characteristics of the participants. As expected, in the MCI group education and cognitive and IADL scores were lower, while age was greater. The total MCSDS score had a mean (standard deviation) of 20.0 (2.6) in the whole sample, 19.6 (2.6) in the NC group and 20.2 (2.5) in the MCI group.

**Table 2:** Main sociodemographic and clinical characteristics of the sample

	NC (*n* = 117)	MCI (*n* = 182)	Total (*n* = 299)	NC vs MCI
Sociodemographics				
Age	78.0 (5.0)	79.2 (5.3)	78.7 (5.2)	**0.044**^b^
Female sex	85 (72.6)	128 (70.3)	213 (71.2)	0.665^d^
Education (yrs)	11.7 (4.3)	10.0 (4.6)	10.7 (4.5)	**0.001**^c^
Income > 1500 €/month^a^	87 (75.0)	119 (66.5)	206 (69.8)	0.119^d^
Cognitive status				
MMSE	28.2 (1.2)	26.6 (2.2)	27.2 (2.0)	**< 0.001**^c^
Global cognition (Z-score)	−0.8 (0.4)	−1.9 (0.7)	−1.5 (0.8)	**< 0.001**^c^
Memory (Z-score)	0.0 (0.5)	−1.1 (0.9)	−0.7 (1.0)	**< 0.001**^b^
Attention/Executive (Z-score)	−3.2 (1.0)	−4.5 (1.2)	−4.0 (1.3)	**< 0.001**^c^
Functional status				
BADL	5.5 (0.5)	5.5 (0.6)	5.5 (0.5)	0.665^c^
IADL	7.1 (1.4)	6.6 (1.5)	6.8 (1.5)	**< 0.001**^c^
Emotional status				
GDS-s	3.3 (2.9)	3.6 (2.9)	3.5 (2.9)	0.319^c^
STPI-TA	18.3 (5.5)	19.1 (5.3)	18.8 (5.4)	0.092^c^
Comorbidity				
CIRS-m	2.4 (1.3)	2.6 (1.5)	2.5 (1.4)	0.553^c^

**Legend**

Continuous variables expressed as mean (standard deviation) and categorical variables as number (percentage). ^a^ Income data missing for 4 participants (1 NC, 3 MCI), ^b^ Student’s t-test, ^c^ Mann-Whitney’s U-test, ^d^ Chi-squared test. Statistically significant results are shown in bold typeface. Abbreviations: *NC* Normal Cognition; *MCI* Mild Cognitive Impairment; *MMSE* Mini Mental State Examination (score range 0–30, higher scores indicate better cognitive functioning); *BADL* Basic Activities of Daily Living (score range 0–6, higher scores indicate greater functional independence); *IADL* Instrumental Activities of Daily Living (score range 0–8, higher scores indicate greater functional independence); *GDS-s* short Geriatric Depression Scale (score range 0–15, higher scores indicate greater depressive symptoms); *STPI-TA* State-Trait Personality Inventory Trait Anxiety subscale (score range 10–40, higher scores indicate greater anxiety symptoms); *CIRS-m* Cumulative Illness Rating Scale comorbidity (score range 0–13, higher scores indicate greater comorbidity)

### Psychometric properties of the eight-item MCSDS

The psychometric properties of the eight-item MCSDS were primarily evaluated across the whole sample because both subjects with NC and MCI (i.e. without dementia) would be expected to be able to understand and answer the questionnaire and pooling data maximises statistical power for reliability and PC analyses [73, 74].

All participants completed the questionnaire and there were no missing answers. There were only 22 “don’t know” answers overall and only 15 participants (i.e. 5% of the sample) responded to the questionnaire by giving at least one “don’t know” answer.

The internal consistency of the scale was found to be poor: Cronbach’s alpha for the overall sample was 0.42 (for the NC group = 0.35, for the MCI group = 0.46, no significant difference in Cronbach’s alphas between groups according to Feldt’s test [75]: *p* = 0.289). The mean inter-item correlation was also well below the recommended 0.15–0.50 range [55, 56] (i.e. 0.09), meaning that the brevity of the scale was not a reason for the low Cronbach’s alpha.

The PCA extracted three components, as shown in Table 3. Items 3 and 4 loaded highly on component 1, which involves admitting to one’s mistakes and can be interpreted as “Acceptance of responsibility”. Items 1 and 2 loaded highly on component 2, which relates to abidance to moral values and can be labelled “Integrity”. Items 5 and 7 loaded highly on component 3, which reflects compassionate interpersonal behaviour and can be designated as “Kindness towards others”. Orthogonal (varimax) rotation yielded the same factorial structure, in accordance with the observed weak correlations between factors.

**Table 3:** PCA with rotated factor loadings^a^ for the three-component factor solution

MCSDS item	Component 1	Component 2	Component 3
1. Have there been occasions when you took advantage of someone?	−0.06	− **0.87**	0.00
2. Have you sometimes taken unfair advantage of another person?	−0.04	− **0.84**	− 0.02
3. Are you always willing to admit when you make a mistake?	**0.86**	−0.02	−0.02
4. Are you quick to admit making a mistake?	**0.86**	−0.06	0.00
5. Do you sometimes try to get even rather than forgive and forget?	−0.15	−0.17	**0.74**
6. Do you sometimes feel resentful when you don't get your own way?	0.12	−0.27	0.02
7. Are you always courteous, even to people who are disagreeable?	−0.01	0.00	**0.66**
8. Are you always a good listener, no matter whom you are talking to?	0.17	0.18	0.49
Mean (SD)	5.2 (1.4)	5.7 (0.9)	5.0 (1.3)
Eigenvalue	1.7	1.5	1.1
Percentage of variance explained	21.5	18.6	14.4
Mean inter-item correlation	0.53	0.53	0.15
Cronbach’s alpha	0.66	0.65	0.26

**Legend**

^a^ Direct oblimin (oblique) rotation. Factor loadings > |0.60| are shown in bold typeface. Component 1 represents “Acceptance of responsibility”, component 2 “Integrity” and component 3 “Kindness towards others”. Abbreviations: *PCA* Principal Components Analysis; *MCSDS* Marlowe-Crowne Social Desirability Scale; *SD* Standard Deviation

The three-component structure of the scale was replicated in both NC and MCI subjects (see Additional file 2).

Cronbach’s alphas were acceptable (i.e. > 0.6) [60, 76] and mean inter-item correlations were high (i.e. > 0.5) for the first two components. However, for the third component Cronbach’s alpha was low and the mean inter-item correlation was just acceptable.

**Fig. 1:**
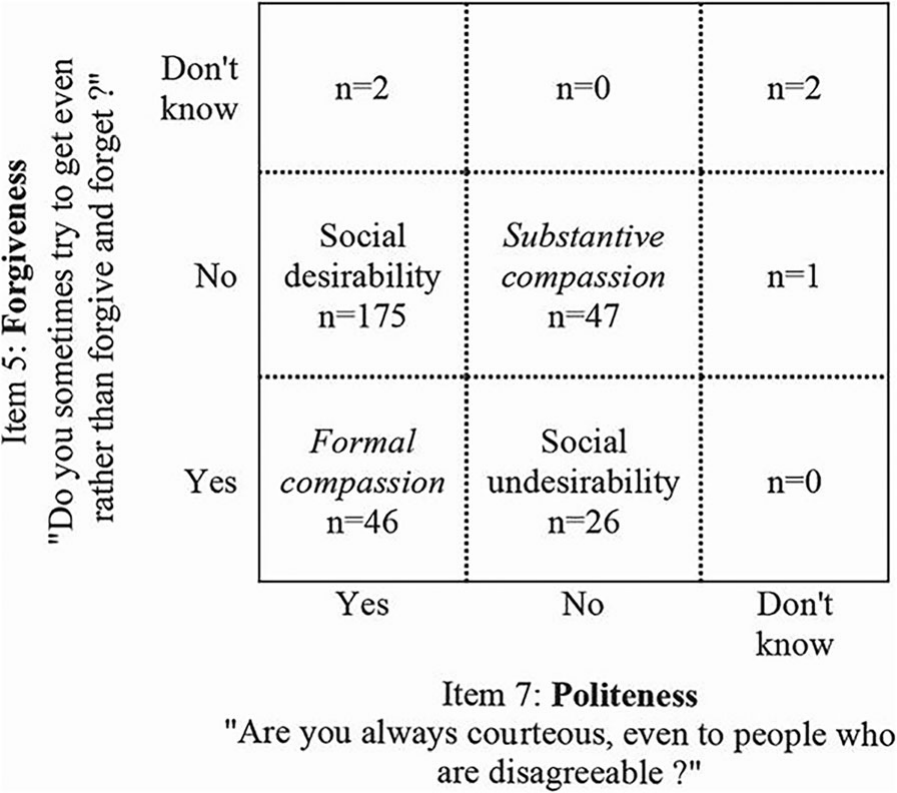
Pattern of responses to the “Kindness towards others” subscale. Congruent responding corresponds to the quadrants labelled Social desirability and Social undesirability. Incongruent responding corresponds to the quadrants labelled Substantive compassion and Formal compassion (in italics)

On further analysis, this could, at least in part, be ascribed to the fact there was a consistent proportion of participants (31%) who responded to the two constituent questions in an incongruent manner (Fig. 1). In fact, several subjects (16%) answered item 5 in a socially desirable way and item 7 in a socially undesirable way, i.e. they reported never trying to get even rather than forgive and forget, but admitted to sometimes not being courteous to people. Viceversa, several others (15%) answered item 5 in a socially undesirable way and item 7 in a socially desirable way, i.e. they acknowledged trying sometimes to get even rather than forgive and forget, but professed to be always courteous, even to disagreeable people.

It thus appeared that the underlying construct of “Kindness towards others” could be separated into two distinct concepts: one of a more substantive nature (i.e. being forgiving but not polite), and one of a more formal nature (i.e. being polite but not forgiving). We performed a binary logistic regression with the nature of the concept (formal vs substantive) as the dependent variable, and sociodemographic and clinical characteristics (age, sex, education, income, global cognition Z-score; GDS-s, STPI-TA and CIRS-m scores) as independent variables which were entered simultaneously in the model. The linearity (Box-Tidwell transformation) and no multicollinearity (VIF < 5) assumptions were satisfied. The only independent predictor of the pattern of incongruent responding was income, i.e. participants with higher income were more likely to report formal rather than substantive kindness towards others (Odds Ratio 3.2, 95% Confidence Interval 1.1–9.5, *p* = 0.038, see Additional file 3).

Since the MCSDS was found to have a multidimensional structure, i.e. it was composed of items tapping different underlying constructs, we believed it would be more conceptually appropriate to conduct the subsequent statistical analyses (see following sections) on scores from the two valid subscales (“Acceptance of responsibility” and “Integrity”) and from the other four individual items. This choice is in line with the general acceptance in the psychometric literature [77–80] that the total score of a scale lacking unidimensionality cannot be meaningfully interpreted because it represents a mixture of several facets. However, for the sake of completeness, we performed the same analyses on the total MCSDS score and found no significant differences in the pattern of results (data not shown).

As far as test-retest reliability was concerned, Gwet’s AC2 ranged from 0.79 to 0.93 (see Additional file 4) and Spearman’s correlation coefficient for the total MCSDS score was 0.76.

### Relationship between social desirability and cognitive functioning

The relationship between social desirability and cognitive functioning was explored in two ways: cognitive functioning was first treated as a dichotomous variable (NC vs MCI categorisation) and then as a continuous variable (cognitive Z-scores). The second approach has two main strengths: it prevents loss of information, increasing the sensitivity of the statistical analysis [81], and it acknowledges the continuum nature of cognitive ageing [82]. Thus, the two groups were first compared on the MCSDS scores and then the correlations (unadjusted and adjusted) between the MCSDS and cognitive Z-scores were evaluated across the whole sample. The results are shown in Tables 4 and 5.

**Table 4:** MCSDS scores in the two cognitive groups

MCSDS	NC (*n* = 117)	MCI (*n* = 182)	*P*-value^a^
Component 1	5.0 (1.5)	5.3 (1.3)	0.105
Component 2	5.6 (0.9)	5.7 (0.9)	0.488
Item 5	2.4 (0.9)	2.6 (0.8)	0.254
Item 6	1.4 (0.8)	1.3 (0.7)	0.312
Item 7	2.4 (0.9)	2.6 (0.8)	**0.013**
Item 8	2.7 (0.7)	2.8 (0.6)	0.475

**Legend**

Scores expressed as mean (standard deviation). ^a^ Mann-Whitney’s U-test. Statistically significant results are shown in bold typeface. Abbreviations: *MCSDS* Marlowe-Crowne Social Desirability Scale; *NC* Normal Cognition; *MCI* Mild Cognitive Impairment

**Table 5:** Spearman’s correlations between MCSDS and cognitive function scores

	Cognitive Z-score					
	Global cognition		Memory		Attention/Executive	
MCSDS	Simple	Partial	Simple	Partial	Simple	Partial
Component 1	−0.11 (0.067)	− 0.11 (0.069)	− 0.07 (0.215)	− 0.05 (0.432)	−0.10 (0.099)	−0.10 (0.078)
Component 2	−0.07 (0.239)	− 0.05 (0.430)	−0.03 (0.581)	− 0.02 (0.759)	−0.04 (0.550)	− 0.01 (0.870)
Item 5	−0.05 (0.356)	− 0.04 (0.531)	−0.06 (0.338)	− 0.06 (0.353)	−0.05 (0.352)	−0.04 (0.462)
Item 6	0.05 (0.414)	0.00 (0.933)	−0.03 (0.598)	−0.05 (0.451)	0.06 (0.331)	0.02 (0.705)
Item 7	−0.15 (**0.009**)	−0.12 (**0.049**)	− 0.15 (**0.010**)	−0.11 (0.070)	− 0.16 (**0.005)**	−0.14 **(0.017)**
Item 8	−0.08 (0.194)	−0.08 (0.175)	− 0.06 (0.332)	−0.05 (0.406)	− 0.05 (0.349)	−0.06 (0.286)

**Legend**

Correlations expressed as correlation coefficient (*P*-value). Partial correlations controlling for age, sex, education, income and GDS-s, STPI-TA and CIRS-m scores. Statistically significant results are shown in bold typeface. Abbreviations: *MCSDS* Marlowe-Crowne Social Desirability Scale; *GDS-s* short Geriatric Depression Scale; *STPI-TA* State-Trait Personality Inventory Trait Anxiety subscale; *CIRS-m* Cumulative Illness Rating Scale comorbidity

The higher score of MCI subjects on item 7 of the MCSDS was statistically significant, but not clinically relevant. Item 7 of the MCSDS exhibited significant, albeit weak, negative correlations with all cognitive Z scores on bivariate testing (r_s_ < |0.3|). These correlations were retained on multivariate testing, after partialling out the effects of sociodemographic and clinical variables, with the Z score for memory achieving borderline statistical significance.

When the correlation analyses were performed separately in the NC and MCI subjects no significant differences were found between groups by Fisher’s r to z transformation test (see Additional file 5).

### Relationship between social desirability and psychological symptoms

The relationship between social desirability and symptoms of depression and anxiety was investigated in a three-step process.

First, we calculated Spearman’ s bivariate correlations between each of the GDS-s and STPI-TA scores and the six MCSDS scores across the whole sample. The results are shown in Table 6. The GDS-s scale was found to have significant, albeit weak (r_s_ < |0.3|), negative correlations with the “Acceptance of responsibility” subscale and items 5 and 6 of the MCSDS. The STPI-TA scale was found to have significant/borderline significant, albeit weak (r_s_ < |0.3|), negative correlations with the “Acceptance of responsibility” subscale and item 6 of the MCSDS. The correlation between the GDS-s and STPI-TA scales was, instead, moderate and positive (r _s_ = 0.53, *p* < 0.001). When the correlation analyses were performed separately in the NC and MCI subjects no significant differences were found between groups by Fisher’s r to z transformation test (see Additional file 6).

**Table 6:** Simple Spearman’s correlations between MCSDS and GDS-s and STPI-TA scores

MCSDS	GDS-s	STPI-TA
Component 1	− 0.13 (**0.031**)	− 0.11 (0.059)
Component 2	−0.09 (0.120)	− 0.03 (0.637)
Item 5	−0.12 (**0.038**)	− 0.04 (0.539)
Item 6	−0.17 (**0.003**)	− 0.25 **(< 0.001**)
Item 7	0.04 (0.518)	−0.08 (0.160)
Item 8	0.00 (0.954)	−0.06 (0.301)

**Legend**

Correlations expressed as correlation coefficient (*P*-value). Statistically significant results are shown in bold typeface. Abbreviations: *MCSDS* Marlowe-Crowne Social Desirability Scale; *GDS-s* short Geriatric Depression Scale; *STPI-TA* State-Trait Personality Inventory Trait Anxiety subscale

We then employed multiple linear regression over the whole sample in order to investigate whether social desirability was a predictor of psychological well-being after controlling for sociodemographic and clinical characteristics. We fitted separate models for each of the two mental health scales and for each of the six MCSDS scores (full model). Thus, the dependent variable was either the GDS-s score (first six models) or the STPI-TA score (last six models) and the independent variables were the individual MCSDS score (the variable of interest) as well as the potential confounders: age, sex, education, income, global cognitive Z score, comorbidity, GDS-score (last six models) and STPI-TA score (first six models).

Lastly, for each regression we also computed a reduced model, without the MCSDS score, to quantify the amount of additional variance explained by the inclusion of the MCSDS score in the model, via the change in R squared statistic. The lack of significant group by MCSDshow [?A3B2 h=0pt,128?]S score interactions rendered it appropriate to collapse NC and MCI subjects into a single group. The results are summarised in Table 7. Regression analyses in which cognition was modelled as a dichotomous variable (NC vs MCI group) rather than a continuous one produced the same results (see Additional file 7).

**Table 7:** MCSDS scores as predictors of depressive and anxiety symptoms

	Dependent variable							
	GDS-s^a^				STPI-TA^b^			
Predictors	β (95% CI)	*P*-value	R^2†^	ΔR^2‡^	β (95% CI)	*P*-value	R^2†^	ΔR^2‡^
Component 1	−0.10 (− 0.30, 0.10)	0.325	0.372	0.002	−0.42 (− 0.79, − 0.04)	**0.032**	0.359	**0.010**
Component 2	−0.13 (− 0.41, 0.16)	0.379	0.372	0.002	0.04 (−0.51, 0.59)	0.879	0.348	0.000
Item 5	−0.25 (− 0.57, 0.07)	0.120	0.375	0.005	0.09 (−0.52, 0.70)	0.767	0.348	0.000
Item 6	−0.05 (− 0.42, 0.32)	0.780	0.370	0.000	−1.00 (−1.68, − 0.31)	**0.004**	0.367	**0.018**
Item 7	0.20 (−0.12, 0.52)	0.216	0.373	0.003	−0.73 (−1.33, − 0.13)	**0.017**	0.361	**0.013**
Item 8	0.14 (−0.30, 0.57)	0.540	0.371	0.001	−0.59 (−1.41, 0.24)	0.163	0.353	0.004

**Legend**

Multiple linear regression with GDS-s and STPI-TA scores as dependent variables. Each row represents a separate model (see text). Only the MCSDS scores are shown as predictors. ^a^ Multiple linear regression with age, sex, education, income, global cognition Z-score, CIRS-m score, STPI-TA score and individual MCSDS scores as predictors (full model). ^b^ Multiple linear regression with age, sex, education, income, global cognition Z-score, CIRS-m score, GDS-s score and individual MCSDS scores as predictors (full model). ^†^ R^2^ for the full model. ^‡^ Change in R^2^ from the reduced model (all predictors except the MCSDS individual score) to the full model. Statistically significant results are shown in bold typeface. Abbreviations: *MCSDS* Marlowe-Crowne Social Desirability Scale; *GDS-s* short Geriatric Depression Scale; *STPI-TA* State-Trait Personality Inventory Trait Anxiety subscale; *β* unstandardised regression coefficient; *CI* Confidence Interval; *CIRS-m* Cumulative Illness Rating Scale comorbidity

The “Acceptance of responsibility” subscale and items 6 and 7 of the MCSDS were significant independent predictors of the STPI-TA score. However, socially desirable responding accounted only for a very small proportion (less than 2%) of additional variance in the STPI-TA score after controlling for sociodemographic and clinical characteristics.

## Discussion

In the current study we investigated the psychometric properties of the eight-item MCSDS in geriatric outpatients without dementia as well as the relationship of social desirability with both cognitive functioning and self-reported psychological symptoms. The high prevalence of MCI in the study (61%) is worthy of specific comment. The percentage of older adults classified as MCI has been consistently shown to vary widely across studies due to several methodological factors such as recruitment source, type and number of tests used to assess cognition, and operationalisation of the MCI criteria [83]. In our case, the high prevalence of MCI in the sample could have two possible explanations. First, the diagnosis of MCI relied on an extensive neuropsychological battery, included all MCI subtypes and was based on somewhat lenient criteria (10th percentile cut-off in at least one cognitive test, no requirement for subjective cognitive impairment or intact IADL). Second, the study was conducted in an outpatient clinic setting. The latter is presumably the most likely explanation, since the prevalence of MCI, across different operational definitions, ranges from 3 to 42% in community-based studies [84] and from 40 to 84% in specialty outpatient clinics [85, 86].

### Psychometric properties of the eight-item MCSDS

The eight-item MCSDS displayed poor internal consistency, in terms of Cronbach’s alpha and mean inter-item correlation, because it lacked unidimensionality, i.e. it did not measure a single construct [87]. Indeed, on PCA it was found to have a multidimensional structure. This in line with reports from other authors investigating both short [17, 88] and full [89, 90] forms of the MCSDS.

In particular, three components were identified: “Acceptance of responsibility”, “Integrity” and “Kindness towards others”. The first component involved admitting to one’s mistakes. The second component reflected abidance to moral values. The third component could be conceptualised as measuring two different constructs: one of a more substantive nature, relating to empathy (i.e. forgiveness but not politeness), and one of a more formal nature, linked to social etiquette (i.e. politeness but not forgiveness).

Interestingly, higher income was an independent predictor of formal rather than substantive “Kindness towards others”. Since income is an indicator of socioeconomic status, this finding fits in nicely with the literature. In fact, there is evidence that people from different social strata endorse different sets of values. Individuals who are higher in social class are more likely to attach importance to good manners [91]. Also, in their moral judgments, they have been shown to prioritise the domain of respect rather than that of no harm to others, while the reverse has been found to be true for individuals who are lower in social standing [92].

Although education is also a proxy for socioeconomic status, it was not found to have the same effect as income. This is probably so because of the geriatric context of the study. In fact, education is mainly a measure of early-life (received) socioeconomic status, while income is an accurate index of late-life (actual) socioeconomic status [93].

Lastly, the test-retest reliability of the MCSDS at one month was found to be substantial to excellent (Gwet’s AC2 ≥ 0.8) [94]. Spearman’s correlation coefficient for the total MCSDS score was excellent (r_s_ = 0.8) [65] and within the higher end of the 0.4 to 0.9 range reported by other studies [67].

### Relationship between social desirability and cognitive functioning

There was a statistically significant but not clinically relevant difference in social desirability between the NC and MCI groups, with the MCI subjects scoring slightly higher on item 7 of the MCSDS (“I am always courteous, even to disagreeable people”). Along the same lines, item 7 of the MCSDS exhibited significant negative, albeit weak (r_s_ < |0.3|), Spearman’s correlations with the Z scores for global cognition, memory and attention/executive functioning, even after controlling for potential confounders. Thus, the observed marginal association between social desirability and cognitive functioning was confined to item 7 of the MCSDS, which pertains to politeness. This result seems to resonate with a handful of case studies and case series in the area of sociolinguistic research. They have noted that, in the conversation of people with dementia, social politeness strategies are retained [95–97] or indeed enhanced [98, 99], supposedly to mask cognitive symptoms that would damage their social persona [96, 98, 99].

However, given their small effect size, our findings provide no consistent support to the hypothesis that cognitive impairment increases socially desirable responding. It is possible that our sample of geriatric outpatients experienced little stigmatisation due to the MCI label since it has been reported that social discrimination positively correlates with the severity of the cognitive disorder [100] and that in “traditional” countries like Italy less stigma is attached to cognitive impairment than elsewhere in Europe [12].

Also, there was no evidence in favour of the alternative hypothesis that cognitive impairment could decrease socially desirable responding because of deficits in social cognition. Although impairment in social cognition has recently gained attention in MCI [13, 14], studies have primarily relied on theory of the mind (ToM) tasks. It is recognised that ToM tasks gauge only a specific aspect of social cognition (i.e. understanding the mental states of others) [101]. It is also accepted that such laboratory-based measures may overstate the difficulties encountered by older subjects in real-life social interactions, in which a meaningful context may bring about efficient compensation [102]. Thus, it could be speculated that more naturalistic, everyday social skills, including the ability to edit responses in terms of their social desirability implications, could be preserved in MCI.

### Relationship between social desirability and psychological symptoms

On correlation analysis there were some significant negative correlations between social desirability and symptoms of depression and anxiety. Although such correlations were weak (r_s_ < |0.3|) and scattered across items of the MCSDS, their direction is in accordance with a large body of literature demonstrating that greater social desirability is associated with higher scores on measures of psychological well-being (e.g. [18, 20, 21, 103]). Likewise, in line with previous research, the correlations between the MCSDS and the well-being scales were weaker than those between the two well-being scales [104].

When multiple linear regression was used to quantify the effect of socially desirable responding on self-rated mental health, after controlling for a number of confounders, we found that social desirability had a statistically significant association with anxiety but not depressive symptoms. Since it would be reasonable to expect the relationship between social desirability and psychological well-being to be more manifest for mental health symptoms that are more prone to stigma, it could be conjectured that anxiety carries a greater stigma burden than depression. As a matter of fact, older adults have been shown to hold more stigmatising attitudes towards their peers with anxiety, whom they perceive as responsible for their condition, than with depression [105]. This is likely to arise from the common misconception - by the public [105, 106], health professionals [107] and depression sufferers themselves [108] – that depression is a normal part of ageing. In any case, such result appears to have little practical relevance, given that socially desirable responding uniquely explained only a small amount of additional variance in anxiety symptoms (i.e. less than 2%) above and beyond sociodemographic and clinical characteristics.

The inconsequential relationship between the MCSDS and the well-being scales documented by the current study is consonant with reports from other authors [21, 22, 24, 25, 104] and contributes to the ongoing debate in the literature on whether socially desirable responding can influence self-reported measures of psychological well-being. In our sample of geriatric outpatients, social desirability had a minimal association with subjective measures of psychological symptoms.

### Strengths and limitations

The main strengths of the study include its novelty, the use of a comprehensive neuropsychological assessment to characterise individuals as NC or MCI, and the fairly large sample size.

Some limitations must also be acknowledged. First, the cross-sectional design of the study does not allow causal inference. Second, even if the statistical analyses were controlled for a number of potential confounders, the risk of residual confounding cannot be excluded. Third, the mode of administration of the questionnaire (by a one-to-one interview rather than by self-completion) involved a social interaction and is likely to have increased socially desirable responding [3, 109]. Although several studies have reported no differences between interviewer- and self-administered questionnaire modes in the type of response to sensitive issues [109], further research is warranted to determine whether our results would hold true if the questionnaires were filled in by the participants themselves. Nonetheless, a few remarks should be made. In geriatric practice, self-report questionnaires, including those on mental health like the GDS-s (e.g. [110]) and the STPI-TA (e.g. [50]) are often read out to the respondents because this method carries advantages in older adults: it is suitable for subjects with physical impairments (e.g. visual or motor) as well as low literacy, it is less cognitively demanding in the presence of age-related cognitive decline, and it enhances item response rates since the interviewer can maintain motivation, provide clarification and probe for responses [109]. Indeed, survey research has shown that older adults prefer interviewer-administered modes over self-administered modes [111, 112]. Within this context, for the sake of comparability, the MCSDS would also have to be delivered by an interviewer, and this may be the reason why most research on social desirability in older age [20–23] has employed a similar strategy. Fourth, we used a mainly binary, eight-item MCSDS to assess social desirability and the sample was somewhat homogeneous in its tendency to score towards the higher end of the range. It is possible that a short MCSDS with a Likert-format (e.g. [113]) could have encouraged more diverse responding, improving internal consistency [114] and providing a more nuanced insight into the magnitude of social desirability. Still, research on older subjects suggests that increasing response options can lead to confusion without increasing response variability [115]. Fifth, we supposed that social desirability in MCI could be either increased or decreased, being potentially affected by stigma and loss of social skills respectively. Since the study was not designed to investigate specific underpinnings of social desirability in cognitive impairment, we did not assess perceived stigma or social cognitive abilities. However, it should be noted that scales that measure stigma are per se subject to the bias of socially desirable responding [116] and this could deeply confound any association between perceived stigma and social desirability (e.g. if subjects with cognitive impairment display greater social desirability because they experience greater stigma they might also be more prone to deny stigma). Moreover, although we did not use specific tests of social cognition, we investigated correlations between MCSDS items and Z-scores for memory and attention/executive functioning. Sixth, we did not correct for multiple testing, so inflation of type I error was not controlled for. Positive findings will therefore need to be confirmed by further studies. Finally, we discussed socially desirable responding as an intentional misrepresentation of the self. This is in keeping with the notion, prevalent in health research, that social desirability is a common source of bias in studies involving self-report measures [117], and with most of the literature on social desirability in older age [20–23]. Yet, we recognise that the stylistic (i.e. response bias) versus substantive (i.e. stable personality trait) nature of social desirability, and its implications for personality assessment, have long been (e.g. [118]) and still are (e.g. [1, 119]) a matter of debate. The controversy is primarily due to the paucity of studies including an external, objective criterion as benchmark (e.g. ratings from informants) and their conflicting results (e.g. [120–122]). Our study did not aim to address this issue since the topic of social desirability is salient to geriatric practice regardless of interpretation [8].

## Conclusions

This study is the first to use the eight-item MCSDS in a sample of geriatric outpatients with and without MCI. The scale was found to have a multidimensional structure, including three main subscales: “Acceptance of responsibility”, “Integrity” and “Kindness towards others”. Internal consistency was acceptable for the first two subscales, but not for the third one. In fact, the “Kindness towards others” construct appeared to comprise two distinct concepts of compassionate behaviour - formal (i.e. politeness) and substantive (i.e. forgiveness) - with higher income being the only predictor of formal compassion. Test-retest reliability was substantial to excellent. There was no consistent evidence for an association between cognitive deficits and socially desirable responding. Also, social desirability had a marginal relationship with self-rated depressive and anxiety symptoms.

Our results suggest that social desirability need not be a major concern when using questionnaires to assess mental health in geriatric outpatients without dementia.

## Supplementary Information

**Additional file 1:** Eight-item Marlowe-Crowne Social Desirability Scale (MCSDS).

**Additional file 2: Tables S1.** and **S2.** PCA of the MCSDS in the NC and MCI groups.

**Additional file 3: Table S3.** Binary logistic regression for the nature of “Kindness towards others” (formal versus substantive) as dependent variable.

**Additional file 4: Table S4.** Test-retest reliability of the MCSDS after one month in a random sample of participants.

**Additional file 5: Tables S5.** to **S7.** Spearman’s correlations between MCSDS and cognitive function scores in the NC and MCI groups and their comparison.

**Additional file 6: Table S8.** Spearman’s correlations between MCSDS and GDS-s and STPI-TA scores in the NC and MCI groups and their comparison.

**Additional file 7: Table S9.** Multiple linear regression with MCSDS scores as predictors of depressive and anxiety symptoms with cognitive status modelled as a dichotomous variable (NC vs MCI).

## Data Availability

The datasets used and/or analysed during the current study are available from the corresponding author on reasonable request.

## Abbreviations

AC: Agreement Coefficient
BADL: Basic Activities of Daily Living
CIRS-m: Cumulative Illness Rating Scale comorbidity
GDS-s: short Geriatric Depression Scale
IADL: Instrumental Activities of Daily Living
MCSDS: Marlowe-Crowne Social Desirability Scale
MCI: Mild Cognitive Impairment
MMSE: Mini Mental State Examination
NC: Normal Cognition
PCA: Principal Components Analysis
STPI-TA: State Trait Personality Inventory Trait Anxiety subscale
ToM: Theory of the Mind
