# Correction to: Socially desirable responding in geriatric outpatients with and without mild cognitive impairment and its association with the assessment of self-reported mental health

**DOI:** 10.1186/s12877-021-02504-3

**Published:** 2021-11-22

**Authors:** Paola Nicolini, Carlo Abbate, Silvia Inglese, Daniela Mari, Paolo D. Rossi, Matteo Cesari

**Affiliations:** 1 Geriatric Unit, Fondazione IRCCS Ca’ Granda Ospedale Maggiore Policlinico, Milan, Italy; 2 IRCCS Fondazione don Carlo Gnocchi, Milan, Italy; 3 Università degli Studi di Milano, Milan, Italy

**Correction to: BMC Geriatrics 21, 494 (2021)**

**https://doi.org/10.1186/s12877-021-02435-z**

After publication of this article [1], the authors reported that in the Results section, in the sentence beginning ‘The lack of significant...’, the term ‘MCSDshow [?A3B2 h = 0 pt.,128?]S ‘should have read ‘MCSDS’. Moreover, some small formatting errors have been corrected.

The original article [1] has been updated.
