# Supplementary material for: Socially desirable responding in geriatric outpatients with and without mild cognitive impairment and its association with the assessment of self-reported mental health
Source: BMC Geriatr. 2021 Sep 15;21:494. doi: 10.1186/s12877-021-02435-z (PMC8442330; doi:10.1186/s12877-021-02435-z)
Supplement: Supplementary file 1 — Additional file 1: Eight-item Marlowe-Crowne Social Desirability Scale (MCSDS). [file 12877_2021_2435_MOESM1_ESM.docx]

**Eight-item Marlowe-Crowne Social Desirability Scale (MCSDS)**

1. Have there been occasions when you took advantage of someone?

YES NO DON'T KNOW/NO ANSWER

2. Have you sometimes taken unfair advantage of another person?

YES NO DON'T KNOW/NO ANSWER

3. Are you always willing to admit when you make a mistake?

YES NO DON'T KNOW/NO ANSWER

4. Are you quick to admit making a mistake?

YES NO DON'T KNOW/NO ANSWER

5. Do you sometimes try to get even rather than forgive and forget?

YES NO DON'T KNOW/NO ANSWER

6. Do you sometimes feel resentful when you don't get your own way?

YES NO DON'T KNOW/NO ANSWER

7. Are you always courteous, even to people who are disagreeable?

YES NO DON'T KNOW/NO ANSWER

8. Are you always a good listener, no matter whom you are talking to?

YES NO DON'T KNOW/NO ANSWER
