# Supplementary material for: Socially desirable responding in geriatric outpatients with and without mild cognitive impairment and its association with the assessment of self-reported mental health
Source: BMC Geriatr. 2021 Sep 15;21:494. doi: 10.1186/s12877-021-02435-z (PMC8442330; doi:10.1186/s12877-021-02435-z)
Supplement: Supplementary file 2 — Additional file 2: Tables S1. and S2. PCA of the MCSDS in the NC and MCI groups. [file 12877_2021_2435_MOESM2_ESM.docx]

**Table S1**. PCA with rotated factor loadings^a^ for the three-component factor solution in the NC group (n = 117)

| MCSDS item | Component 1 | Component 2 | Component 3 |
| --- | --- | --- | --- |
| 1. Have there been occasions when you took advantage of someone? | 0.01 | **-0.84** | -0.14 |
| 2. Have you sometimes taken unfair advantage of another person? | -0.01 | **-0.83** | 0.19 |
| 3. Are you always willing to admit when  you make a mistake? | **0.84** | -0.02 | 0.19 |
| 4. Are you quick to admit making a  mistake? | **0.85** | 0.03 | -0.13 |
| 5. Do you sometimes try to get even rather than forgive and forget? | -0.16 | -0.08 | 0.34 |
| 6. Do you sometimes feel resentful when  you don't get your own way? | 0.28 | -0.15 | -0.40 |
| 7. Are you always courteous, even to people who are disagreeable? | 0.23 | -0.23 | **0.72** |
| 8. Are you always a good listener, no matter whom you are talking to? | 0.23 | 0.26 | 0.59 |
| Mean (SD) | 5.0 (1.5) | 5.6 (0.9) | 2.4 (0.9) |
| Eigenvalue | 1.7 | 1.5 | 1.2 |
| Percentage of variance explained | 20.7 | 19.2 | 15.0 |
| Mean inter-item correlation | 0.53 | 0.46 | - |
| Cronbach's alpha | 0.67 | 0.54 | - |

**Legend**

^a^ Direct oblimin (oblique) rotation. Factor loadings > |0.60| are shown in bold typeface. Component 1 represents "Acceptance of responsibility", component 2 "Integrity" and component 3 "Kindness towards others". Abbreviations: PCA, Principal Components Analysis; NC, Normal Cognition; MCSDS, Marlowe-Crowne Social Desirability Scale; SD, Standard Deviation.

**Table S2**. PCA with rotated factor loadings^a^ for the three-component factor solution in the MCI group (n = 182)

| MCSDS item | Component 1 | Component 2 | Component 3 |
| --- | --- | --- | --- |
| 1. Have there been occasions when you took advantage of someone? | -0.03 | **-0.88** | -0.03 |
| 2. Have you sometimes taken unfair advantage of another person? | -0.01 | **-0.88** | -0.07 |
| 3. Are you always willing to admit when  you make a mistake? | **0.87** | -0.03 | -0.21 |
| 4. Are you quick to admit making a  mistake? | **0.86** | -0.06 | 0.00 |
| 5. Do you sometimes try to get even rather than forgive and forget? | 0.05 | -0.13 | **0.76** |
| 6. Do you sometimes feel resentful when  you don't get your own way? | 0.06 | -0.27 | 0.24 |
| 7. Are you always courteous, even to people who are disagreeable? | -0.09 | 0.14 | **0.76** |
| 8. Are you always a good listener, no matter whom you are talking to? | 0.29 | 0.08 | 0.17 |
| Mean (SD) | 5.3 (1.3) | 5.7 (0.9) | 5.2 (1.3) |
| Eigenvalue | 1.4 | 1.8 | 1.2 |
| Percentage of variance explained | 17.9 | 23.1 | 15.2 |
| Mean inter-item correlation | 0.53 | 0.59 | 0.23 |
| Cronbach's alpha | 0.65 | 0.72 | 0.38 |

**Legend**

^a^ Direct oblimin (oblique) rotation. Factor loadings > |0.60| are shown in bold typeface. Component 1 represents "Acceptance of responsibility", component 2 "Integrity" and component 3 "Kindness towards others". Abbreviations: PCA, Principal Components Analysis; MCI, Mild Cognitive Impairment; MCSDS, Marlowe-Crowne Social Desirability Scale; SD, Standard Deviation.
