# Supplementary material for: Socially desirable responding in geriatric outpatients with and without mild cognitive impairment and its association with the assessment of self-reported mental health
Source: BMC Geriatr. 2021 Sep 15;21:494. doi: 10.1186/s12877-021-02435-z (PMC8442330; doi:10.1186/s12877-021-02435-z)
Supplement: Supplementary file 3 — Additional file 3: Table S3. Binary logistic regression for the nature of “Kindness towards others” (formal versus substantive) as dependent variable. [file 12877_2021_2435_MOESM3_ESM.docx]

**Table S3**. Binary logistic regression for the nature of "Kindness towards others" (formal versus substantive) as dependent variable (n = 93)

| Independent variable | Odds Ratio | 95% CI | P-value |
| --- | --- | --- | --- |
| Age | 0.95 | 0.86, 1.06 | 0.345 |
| Female sex | 2.04 | 0.71, 5.88 | 0.186 |
| Education (years) | 0.99 | 0.89, 1.11 | 0.904 |
| Income > 1500 €/month | 3.18 | 1.06, 9.50 | **0.038** |
| Global cognition (Z-score) | 0.92 | 0.50, 1.69 | 0.781 |
| GDS-s | 1.14 | 0.96, 1.36 | 0.133 |
| STPI-TA | 0.94 | 0.85, 1.04 | 0.222 |
| CIRS-m | 1.09 | 0.77, 1.54 | 0.619 |

**Legend**

All independent variables entered simultaneously in the model. Statistically significant results are shown in bold typeface. Abbreviations: CI, Confidence Interval; GDS-s, short Geriatric Depression Scale; STPI-TA, State-Trait Personality Inventory Trait Anxiety Subscale; CIRS-m, Cumulative Illness Rating Scale comorbidity.
