# Supplementary material for: Socially desirable responding in geriatric outpatients with and without mild cognitive impairment and its association with the assessment of self-reported mental health
Source: BMC Geriatr. 2021 Sep 15;21:494. doi: 10.1186/s12877-021-02435-z (PMC8442330; doi:10.1186/s12877-021-02435-z)
Supplement: Supplementary file 4 — Additional file 4: Table S4. Test-retest reliability of the MCSDS after one month in a random sample of participants. [file 12877_2021_2435_MOESM4_ESM.docx]

**Table S4**. Test-retest reliability of the MCSDS after one month in a random sample of participants (n = 50)

| MCSDS | Gwet's AC2^a^ |
| --- | --- |
| Total score | 0.89 |
| Component 1 | 0.82 |
| Component 2 | 0.93 |
| Item 5 | 0.82 |
| Item 6 | 0.79 |
| Item 7 | 0.84 |
| Item 8 | 0.93 |

**Legend**

^a^ Linear-weighted Gwet’s AC2. Abbreviations: MCSDS, Marlowe-Crowne Social Desirability Scale; AC2, Agreement Coefficient (second-order).
