# Supplementary material for: Socially desirable responding in geriatric outpatients with and without mild cognitive impairment and its association with the assessment of self-reported mental health
Source: BMC Geriatr. 2021 Sep 15;21:494. doi: 10.1186/s12877-021-02435-z (PMC8442330; doi:10.1186/s12877-021-02435-z)
Supplement: Supplementary file 5 — Additional file 5: Tables S5. to S7. Spearman’s correlations between MCSDS and cognitive function scores in the NC and MCI groups and their comparison. [file 12877_2021_2435_MOESM5_ESM.docx]

**Table S5**. Spearman's correlations between MCSDS and cognitive function scores in the NC group (n = 117)

|  | Cognitive Z-score | | | | | |
| --- | --- | --- | --- | --- | --- | --- |
|  | Global cognition | | Memory | | Attention/Executive | |
| MCSDS | Simple | Partial | Simple | Partial | Simple | Partial |
| Component 1 | -0.17  (0.066) | -0.12  (0.226) | -0.07  (0.488) | -0.03  (0.789) | -0.14  (0.125) | -0.13  (0.195) |
| Component 2 | -0.01  (0.922) | 0.02  (0.839) | -0.01  (0.887) | 0.02  (0.818) | 0.14  (0.128) | 0.19  (**0.047**) |
| Item 5 | 0.04  (0.688) | 0.00  (0.970) | 0.09  (0.354) | 0.07  (0.460) | 0.00  (0.997) | -0.03 (0.782) |
| Item 6 | 0.05  (0.599) | 0.04  (0.684) | -0.13  (0.150) | -0.14  (0.141) | 0.15  (0.107) | 0.15  (0.131) |
| Item 7 | -0.10  (0.297) | -0.07  (0.475) | -0.03  (0.730) | 0.01  (0.909) | -0.07  (0.482) | -0.04  (0.703) |
| Item 8 | 0.00  (0.994) | 0.00  (0.965) | 0.08  (0.394) | 0.09  (0.332) | -0.07  (0.463) | -0.10  (0.296) |

**Legend**

Correlations expressed as correlation coefficient (P-value). Partial correlations controlling for age, sex, education, income and GDS-s, STPI-TA and CIRS-m scores. Statistically significant results are shown in bold typeface. Abbreviations: MCSDS, Marlowe-Crowne Social Desirability Scale; NC, Normal Cognition; GDS-s, short Geriatric Depression Scale; STPI-TA, State-Trait Personality Inventory Trait Anxiety subscale; CIRS-m, Cumulative Illness Rating Scale comorbidity.

**Table S6**. Spearman's correlations between MCSDS and cognitive function scores in the MCI group (n = 182)

|  | Cognitive Z-score | | | | | |
| --- | --- | --- | --- | --- | --- | --- |
|  | Global cognition | | Memory | | Attention/Executive | |
| MCSDS | Simple | Partial | Simple | Partial | Simple | Partial |
| Component 1 | 0.01  (0.939) | 0.01  (0.889) | 0.01  (0.847) | 0.06  (0.436) | 0.00  (0.973) | 0.02  (0.766) |
| Component 2 | -0.08  (0.298) | -0.04  (0.623) | -0.01  (0.888) | 0.01  (0.903) | -0.08  (0.311) | -0.04  (0.629) |
| Item 5 | -0.06  (0.451) | 0.06  (0.443) | -0.11  (0.158) | -0.06  (0.441) | -0.04  (0.625) | 0.05  (0.484) |
| Item 6 | -0.03  (0.740) | 0.00  (0.975) | -0.07  (0.332) | 0.00  (0.958) | -0.06  (0.401) | -0.01  (0.865) |
| Item 7 | -0.03  (0.711) | 0.05  (0.511) | -0.09  (0.210) | -0.05  (0.519) | -0.07  (0.359) | -0.03  (0.693) |
| Item 8 | -0.09  (0.256) | -0.06  (0.477) | -0.11  (0.131) | -0.06  (0.439) | -0.02  (0.833) | 0.01  (0.866) |

**Legend**

Correlations expressed as correlation coefficient (P-value). Partial correlations controlling for age, sex, education, income and GDS-s, STPI-TA and CIRS-m scores. Abbreviations: MCSDS, Marlowe-Crowne Social Desirability Scale; MCI, Mild Cognitive Impairment; GDS-s, short Geriatric Depression Scale; STPI-TA, State-Trait Personality Inventory Trait Anxiety subscale; CIRS-m, Cumulative Illness Rating Scale comorbidity.

**Table S7**. Fisher's r to z transformation test comparing Spearman's correlations between the NC and MCI groups

|  | Cognitive Z-score | | | | | |
| --- | --- | --- | --- | --- | --- | --- |
|  | Global cognition | | Memory | | Attention/Executive | |
| MCSDS | Simple | Partial | Simple | Partial | Simple | Partial |
| Component 1 | 0.150 | 0.300 | 0.522 | 0.488 | 0.253 | 0.231 |
| Component 2 | 0.575 | 0.640 | 0.987 | 0.917 | 0.076 | 0.063 |
| Item 5 | 0.446 | 0.611 | 0.120 | 0.291 | 0.764 | 0.514 |
| Item 6 | 0.548 | 0.741 | 0.611 | 0.263 | 0.083 | 0.197 |
| Item 7 | 0.574 | 0.333 | 0.620 | 0.623 | 0.981 | 0.955 |
| Item 8 | 0.495 | 0.681 | 0.118 | 0.217 | 0.673 | 0.357 |

Abbreviations: NC, Normal Cognition; MCI, Mild Cognitive Impairment; MCSDS, Marlowe-Crowne Social Desirability Scale.
