# Supplementary material for: Socially desirable responding in geriatric outpatients with and without mild cognitive impairment and its association with the assessment of self-reported mental health
Source: BMC Geriatr. 2021 Sep 15;21:494. doi: 10.1186/s12877-021-02435-z (PMC8442330; doi:10.1186/s12877-021-02435-z)
Supplement: Supplementary file 6 — Additional file 6: Table S8. Spearman’s correlations between MCSDS and GDS-s and STPI-TA scores in the NC and MCI groups and their comparison. [file 12877_2021_2435_MOESM6_ESM.docx]

**Table S8**. Simple Spearman’s correlations between MCSDS and GDS-s and STPI-TA scores in the NC and MCI groups

|  | GDS-s | | Fisher’s  r to z  test | STPI-TA | | Fisher’s  r to z  test |
| --- | --- | --- | --- | --- | --- | --- |
|  | NC  (n = 117) | MCI  (n =182) |  | NC  (n =117) | MCI  (n =182) |  |
| MCSDS |  |  |  |  |  |  |
| Component 1 | -0.04 (0.643) | -0.19 (**0.012**) | 0.236 | -0.07 (0.478) | -0.15 (**0.038**) | 0.470 |
| Component 2 | -0.03 (0.776) | -0.14 (0.064) | 0.365 | 0.02 (0.866) | -0.07 (0.325) | 0.470 |
| Item 5 | -0.04 (0.701) | -0.18 (**0.015**) | 0.237 | -0.09 (0.359) | -0.01 (0.944) | 0.510 |
| Item 6 | -0.04 (0.700) | -0.27  (**< 0.001**) | 0.055 | -0.16 (0.084) | -0.32  **(< 0.001**) | 0.162 |
| Item 7 | 0.03 (0.789) | 0.04 (0.624) | 0.922 | -0.12 (0.214) | -0.07 (0.327) | 0.725 |
| Item 8 | (0.431) | -0.04 (0.584) | 0.351 | -0.08 (0.386) | -0.06 (0.405) | 0.877 |

**Legend**

Correlations expressed as correlation coefficient (P-value). Correlations between the two groups compared with Fisher’s r to z transformation test. Statistically significant results are shown in bold typeface. Abbreviations: MCSDS, Marlowe-Crowne Social Desirability Scale; GDS-s, short Geriatric Depression Scale; STPI-TA, State-Trait Personality Inventory Trait Anxiety subscale;

NC, Normal Cognition; MCI, Mild Cognitive Impairment.
