# Supplementary material for: Socially desirable responding in geriatric outpatients with and without mild cognitive impairment and its association with the assessment of self-reported mental health
Source: BMC Geriatr. 2021 Sep 15;21:494. doi: 10.1186/s12877-021-02435-z (PMC8442330; doi:10.1186/s12877-021-02435-z)
Supplement: Supplementary file 7 — Additional file 7: Table S9. Multiple linear regression with MCSDS scores as predictors of depressive and anxiety symptoms with cognitive status modelled as a dichotomous variable (NC vs MCI). [file 12877_2021_2435_MOESM7_ESM.docx]

**Table S9**. MCSDS scores as predictors of depressive and anxiety symptoms

|  | Dependent variable | | | | | | | |
| --- | --- | --- | --- | --- | --- | --- | --- | --- |
|  | GDS-s^a^ | | | | STPI-TA^b^ | | | |
| Predictors | β  (95% CI) | P-value | R^2†^ | ΔR^2‡^ | β  (95% CI) | P-value | R^2†^ | ΔR^2‡^ |
|  |  |  |  |  |  |  |  |  |
| Component 1 | -0.10  (-0.30, 0.10) | 0.317 | 0.371 | 0.002 | -0.41  (-0.79, -0.04) | **0.032** | 0.358 | **0.010** |
| Component 2 | -0.13  (-0.42, 0.15) | 0.358 | 0.370 | 0.002 | 0.05  (-0.50, 0.60) | 0.856 | 0.348 | 0.000 |
| Item 5 | -0.26  (-0.58, 0.06) | 0.114 | 0.374 | 0.006 | 0.07  (-0.54, 0.68) | 0.822 | 0.348 | 0.000 |
| Item 6 | -0.05  (-0.41, 0.32) | 0.812 | 0.369 | 0.000 | -0.99  (-1.67, -0.31) | **0.005** | 0.366 | **0.018** |
| Item 7 | 0.20  (-0.12, 0.52) | 0.212 | 0.372 | 0.003 | -0.73  (-1.33, -0.13) | **0.017** | 0.361 | **0.013** |
| Item 8 | 0.12  (-0.31, 0.55) | 0.589 | 0.369 | 0.001 | -0.57  (-1.39, 0.26) | 0.176 | 0.352 | 0.004 |

**Legend**

Multiple linear regression with GDS-s and STPI-TA scores as dependent variables. Each row represents a separate model (see text). Only the MCSDS scores are shown as predictors. ^a^ Multiple linear regression with age, sex, education, income, cognitive group (NC vs MCI), CIRS-m score, STPI-TA score and individual MCSDS scores as predictors (full model). ^b^ Multiple linear regression with age, sex, education, income, cognitive group (NC vs MCI), CIRS-m score, GDS-s score and individual MCSDS scores as predictors (full model). ^†^ R^2^ for the full model. ^‡^ Change in R^2^ from the reduced model (all predictors except the MCSDS individual score) to the full model. Statistically significant results are shown in bold typeface. Abbreviations: MCSDS, Marlowe-Crowne Social Desirability Scale; GDS-s, short Geriatric Depression Scale; STPI-TA, State-Trait Personality Inventory Trait Anxiety subscale; β, unstandardised regression coefficient; CI, Confidence Interval; CIRS-m, Cumulative Illness Rating Scale comorbidity.
